# CXCR4 Antagonists as Stem Cell Mobilizers and Therapy Sensitizers for Acute Myeloid Leukemia and Glioblastoma?

**DOI:** 10.3390/biology9020031

**Published:** 2020-02-17

**Authors:** Vashendriya V.V. Hira, Cornelis J.F. Van Noorden, Remco J. Molenaar

**Affiliations:** 1 Department of Genetic Toxicology and Cancer Biology, National Institute of Biology, 1000 Ljubljana, Sloveniar.j.molenaar@amsterdamumc.nl (R.J.M.); 2 Department of Medical Biology, Cancer Center Amsterdam, Amsterdam UMC at the Academic Medical Center, 1105 AZ Amsterdam, The Netherlands; 3 Department of Medical Oncology, Cancer Center Amsterdam, Amsterdam UMC at the Academic Medical Center, 1105 AZ Amsterdam, The Netherlands

**Keywords:** Glioblastoma, glioblastoma stem cells, niches, acute myeloid leukemia, hematopoietic stem cells, bone marrow, C-X-C receptor type 4, stromal-derived factor-1α, plerixafor

## Abstract

Glioblastoma is the most aggressive and malignant primary brain tumor in adults and has a poor patient survival of only 20 months after diagnosis. This poor patient survival is at least partly caused by glioblastoma stem cells (GSCs), which are slowly-dividing and therefore therapy-resistant. GSCs are localized in protective hypoxic peri-arteriolar niches where these aforementioned stemness properties are maintained. We previously showed that hypoxic peri-arteriolar GSC niches in human glioblastoma are functionally similar to hypoxic peri-arteriolar hematopoietic stem cell (HSC) niches in human bone marrow. GSCs and HSCs express the receptor C-X-C receptor type 4 (CXCR4), which binds to the chemoattractant stromal-derived factor-1α (SDF-1α), which is highly expressed in GSC niches in glioblastoma and HSC niches in bone marrow. This receptor–ligand interaction retains the GSCs/HSCs in their niches and thereby maintains their slowly-dividing state. In acute myeloid leukemia (AML), leukemic cells use the SDF-1α–CXCR4 interaction to migrate to HSC niches and become slowly-dividing and therapy-resistant leukemic stem cells (LSCs). In this communication, we aim to elucidate how disruption of the SDF-1α–CXCR4 interaction using the FDA-approved CXCR4 inhibitor plerixafor (AMD3100) may be used to force slowly-dividing cancer stem cells out of their niches in glioblastoma and AML. Ultimately, this strategy aims to induce GSC and LSC differentiation and their sensitization to therapy.

Glioblastoma (World Health Organization grade IV glioma) is the most aggressive and lethal primary brain tumor in adults, with a poor survival of only 20 months after diagnosis despite surgery, radiotherapy, temozolomide chemotherapy and magnetic tumor-treating fields (TTF) in the fittest patient population [1,2,3,4,5]. This poor patient survival is at least partly caused by glioblastoma stem cells (GSCs) that reside in protective niches where they are maintained as slowly-dividing and therapy-resistant cells [6,7,8,9]. Various cell types and tissue structures in the tumor microenvironment enforce the stem cell characteristics on GSCs. These microenvironmental cues enable GSCs to adapt to changing circumstances in the tumor and, as a consequence, GSCs are maintained as quiescent and therapy-resistant cells [10,11].

In our previous studies, we provided evidence that GSC niches in human glioblastoma tumors are functionally similar to hematopoietic stem cell (HSC) niches in normal human bone marrow [12,13]. Both GSC niches and HSC niches are hypoxic and peri-arteriolar where the GSCs/HSCs are localized adjacent to the tunica adventitia of the arteriolar wall [8,12,14,15]. The chemoattractant stromal-derived factor-1α (SDF-1α; also known as CXCL12) is abundantly expressed in GSC/HSC niches for homing of C-X-C receptor type 4 (CXCR4)-positive GSCs/HSCs in their niches [8,12,14,15]. Hypoxia is crucial for the maintenance of slowly-dividing GSCs/HSCs in niches. The transcription factors hypoxia-inducible factor-1α (HIF-1α) and HIF-2α are highly expressed in niches and in turn upregulate expression of stem cell genes, such as CD133 and sex-determining region Y-box factor 2 (SOX2) for GSCs, and CD133 and CD150 for HSCs [12]. In addition, hypoxia upregulates the expression of SDF-1α and its receptor CXCR4 in glioblastoma tumors and bone marrow via HIF-1α [9,15,16,17,18,19]. In Figure 1, a peri-arteriolar HSC niche in bone marrow (Figure 1A,B) and a peri-arteriolar GSC niche in a glioblastoma tumor (Figure 1C,D) are shown. HSCs and GSCs express the stem cell marker CD133 and the SDF-1α receptor CXCR4. The GSCs/HSCs are localized around smooth muscle actin-positive arterioles in niches that contain extracellular SDF-1α [12].

From a clinical perspective, our finding that GSC niches in glioblastoma and HSC niches in bone marrow are functionally similar is relevant as it provides a rationale for the repurposing of hematological drugs to the glioblastoma clinic. One such example is the CXCR4 inhibitor plerixafor (AMD3100). Plerixafor is an FDA-approved drug for patients who undergo an autologous stem cell transplantation as a treatment for multiple myeloma (MM) or non-Hodgkin’s lymphoma. In this setting, plerixafor is used as a HSC mobilizer and optimizes HSC yields after plasmapheresis, which results in a better clinical outcome after stem cell transplantation [20,21,22,23].

In addition, plerixafor is currently in clinical trials for the treatment of hematological malignancies themselves, such as acute myeloid leukemia (AML). The rationale behind this possible therapy is that AML cells can relocate to HSC niches in bone marrow via interactions between receptor CXCR4 and chemoattractant SDF-1α to become slowly-dividing leukemic stem cells (LSCs) that are resistant to chemotherapy. This phenomenon is called ‘’hijacking of HSC niches’’, which causes relapse in AML patients [24,25,26,27,28]. In vitro studies of a preclinical AML model showed that plerixafor effectively mobilizes slowly-dividing and chemotherapy-resistant LSCs out of HSC niches into the peripheral blood, which results in LSC differentiation and, as a consequence, sensitization to chemotherapy [28,29,30]. In a non-randomized phase I/II clinical trial including 52 AML patients, the combination of plerixafor and MEC (mitoxantrone, etoposide and cytarabine) chemotherapy was tested [31,32]. A comparison with a historical control cohort showed that the combination of plerixafor and MEC chemotherapy resulted in two-fold increased numbers of AML cells in the peripheral blood and a more than doubled higher overall complete response rate (46%) [31,32] compared to MEC chemotherapy alone (21%) [33]. A phase I clinical trial with a group of 69 AML patients showed that plerixafor can be safely used in combination with the chemotherapeutic agent decitabine, which resulted in mobilization of LSCs out of HSC niches. Whereas the clinical benefits of plerixafor in this setting are uncertain, this was primarily a proof-of-principle study and the data suggest that the therapeutic strategy was successful, as mobilization of LSCs out of the niches into the peripheral blood was achieved [34].

Our point of view is that inhibition of the receptor CXCR4 is a promising approach to enable GSC/LSC mobilization out of their protective niches, which results in GSC/LSC differentiation and proliferation and thereby sensitization to therapy.

Because of the homology of the hypoxic peri-arteriolar HSC niches in bone marrow and the hypoxic peri-arteriolar GSC niches in glioblastoma tumors, we postulate the hypothesis that plerixafor treatment in combination with standard therapies is beneficial for glioblastoma patients as well. This hypothesis is based on the assumption that plerixafor treatment can mobilize GSCs out of their protective niches, which results in GSC differentiation and their sensitization to radiotherapy and chemotherapy [8,12,13,15]. In a single-arm phase I/II clinical trial including 29 glioblastoma patients, intravenous administration of plerixafor was combined with radiotherapy and temozolomide chemotherapy [35,36]. In this communication, we compare the patient characteristics of this clinical trial with the patient characteristics and survival outcomes of the most recent landmark clinical trial in newly-diagnosed glioblastoma patients [5]. The clinical trials had highly similar inclusion and exclusion criteria. Compared with the historical control cohort, the plerixafor clinical trial included patients who were slightly older, underwent a gross total resection less often and had worse Karnofsky performance scores (all being detrimental prognostic factors [37]) but a higher proportion of patients had a glioblastoma with an *IDH1* mutation (being a positive prognostic factor, Table 1 [37,38,39]).

Taken together, we conclude that the patient population from the plerixafor clinical trial has similar prognostic features compared with the historical control cohort, in which a median overall survival of 16.0 months was achieved after treatment with radiotherapy and temozolomide chemotherapy alone. In comparison, the aforementioned plerixafor clinical trial yielded a median overall survival of 21.3 months [35,36]. This suggests that plerixafor treatment in combination with radiotherapy can prolong glioblastoma patient survival [35,40]. In addition, plerixafor was well tolerated at the highest dose of 400 µg/kg/day with no Common Terminology Criteria for Adverse Events (CTCAE) [41] grade 3 or higher toxicities. The authors of the phase I/II clinical trial postulated the hypothesis that hypoxia-induced SDF-1α secretion after radiotherapy causes infiltration of bone marrow-derived macrophages/monocytes into the glioblastoma tumor via interactions between SDF-1α and CXCR4 and/or CXCR7 receptors that are expressed on the macrophages/monocytes, resulting in vasculogenesis and tumor recurrence [42,43,44]. Therefore, the aim was to block the SDF-1α–CXCR4/CXCR7 pathway using plerixafor to abrogate these phenomena. This strategy is called macrophage exclusion after radiation therapy (MERT) [36]. Elevated plerixafor serum levels, elevated SDF-1α plasma levels and increased numbers of intravascular bone marrow-derived monocytes/macrophages confirmed CXCR4 blockade in the patients [36]. We suggest that CXCR4 inhibition can facilitate the mobilization of GSCs out of their protective niches as well as the mobilization of macrophages out of the tumor, resulting in a better response to radiotherapy and temozolomide chemotherapy.

In this phase I/II clinical trial, the subventricular zone (SVZ) was not irradiated [36]. The SVZ is localized at the border of the lateral ventricles in the cerebrum, where neural stem cells (NSCs) are maintained in niches. The SVZ is also a preferable site for GSCs, as high levels of SDF-1α facilitate homing of CXCR4-positive GSCs in the SVZ, where GSCs are protected from the effects of radiotherapy. In mouse models, application of plerixafor as well as SDF-1α-blocking antibodies sensitized SVZ-residing GSCs to radiotherapy [45,46]. Therefore, we suggest that in clinical trials, the irradiated area should also include the SVZ region after plerixafor treatment to sensitize SVZ-residing GSCs to radiotherapy as well. In agreement with this hypothesis, a phase II clinical trial that is currently recruiting patients will test whole-brain radiotherapy in combination with temozolomide chemotherapy and plerixafor treatment (ClinicalTrials.gov Identifier: NCT03746080).

Multiple studies have shown that CXCR4 is overexpressed in glioblastoma cells, which is associated with increased invasive behavior of glioblastoma cells towards high SDF-1α concentrations [47,48,49,50]. In a study by Yadav et al. (2016), it was shown that human GSCs express high levels of CXCR4 and are attracted towards human brain microvascular endothelial cells that secrete SDF-1α in vitro, and peri-vascular invasion of GSCs was observed in mouse models of glioblastoma. Blocking CXCR4, using plerixafor as well as shRNA-mediated knockdown of CXCR4, downregulated this invasive behavior of GSCs in mice, which resulted in prolonged median survival. In addition, it was demonstrated that CXCR4 inhibition results in increased sensitivity of GSCs to radiotherapy [48]. Thus, CXCR4 inhibition is a promising approach to downregulate GSC invasion and to sensitize GSCs to standard therapies [47,48,49,51].

Besides plerixafor, there are also other CXCR4 antagonists that are promising to mobilize GSCs out of their protective niches. The novel experimental CXCR4 antagonist PRX17756 has been shown to actively penetrate the blood-brain barrier and to accelerate GSC differentiation [52,53]. Another promising CXCR4 antagonist is BL-8040, a small synthetic peptide (14 amino acids in size) with high affinity for CXCR4 [54]. Therefore, PRX17756 and BL-8040 should also be tested in clinical trials in glioblastoma patients in combination with radiotherapy and temozolomide.

The role of CXCR4 inhibition was investigated in mouse models of immunotherapy of solid tumors. BL-8040 was used to mobilize immune progenitor cells out of bone marrow to obtain higher amounts of T-cells in the circulation and a better infiltration of the tumor, resulting in more effective immune therapy. This in vivo study showed that the CXCR4 inhibitor BL-8040 may have anti-tumor effects by increasing tumor infiltration of antigen-specific effector T-cells [54]. Besides the most-studied plerixafor, many other CXCR4 antagonists have been applied and tested in various types of cancer (Table 2).

Besides chemoattraction and binding, the SDF-1α–CXCR4 axis is important for cell survival. GSCs express both SDF-1α and CXCR4 [12], which induces an autocrine loop, resulting in activation of the PI3K–MAPK–ERK1/2 signaling pathway for cell survival [75]. Thus, apart from mobilization of GSCs out of their niches, the abrogation of the SDF-1α–CXCR4 axis may also reduce GSC survival [75].

Bone marrow-derived mesenchymal stem cells (MSCs) are known to be producers of SDF-1α [12], infiltrate glioblastoma tumors and have affinity for CD133-positive GSCs [76,77,78,79]. Our previous studies have shown that CXCR4-positive MSCs are exclusively localized in both HSC niches in normal bone marrow and GSC niches in glioblastoma tumors [12]. Their exact function in glioblastoma tumors is not fully understood. However, our unpublished data suggest that MSCs are involved in attraction and retention of CXCR4-positive GSCs in protective niches. Therefore, plerixafor may also be useful to disrupt interactions between GSCs and MSCs in peri-arteriolar GSC niches.

In conclusion, our hypotheses are that plerixafor and other CXCR4 antagonists are promising as GSC/LSC mobilizers and therapy sensitizers, because they can induce GSC/LSC differentiation into rapidly-dividing progenitor cells that are more vulnerable to chemotherapy and radiotherapy. In addition, SDF-1α–CXCR4 inhibition can reduce the pro-cell survival effects in GSCs/LSCs.

## Figures and Tables

**Figure 1:**
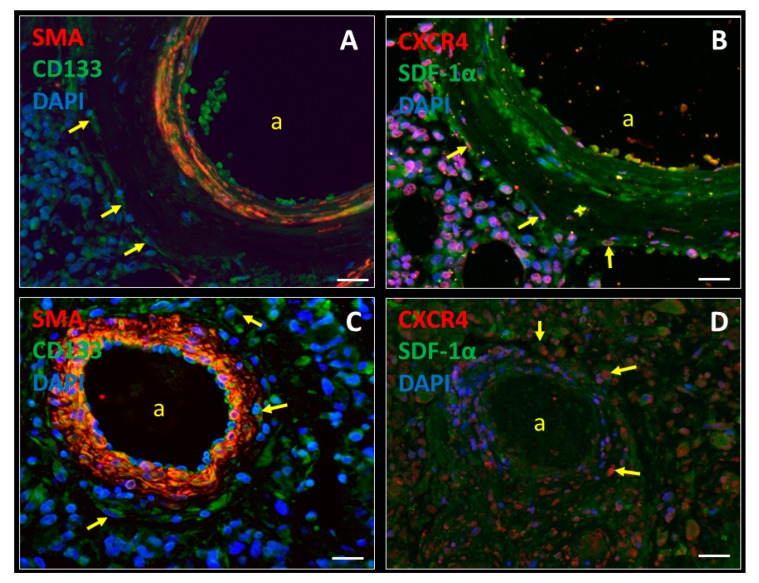
Immunofluorescence staining of a hypoxic peri-arteriolar HSC niche in bone marrow (**A**,**B**) and a hypoxic peri-arteriolar GSC niche in a glioblastoma tumor (**C**,**D**). HSCs (A) and GSCs (C) express the stem cell marker CD133 and are localized around the tunica adventitia and the SMA-positive tunica media of an arteriolar wall (a) (A, C, yellow arrows). Receptor CXCR4 is expressed by HSCs (B) and GSCs (D) in SDF-1α-rich niches (B, D, yellow arrows). SDF-1α is expressed extracellularly and is also expressed by endothelial cells and smooth muscle cells in the tunica media of the arteriolar wall in both HSC niches and GSC niches (B, D). DAPI (blue) is used for nuclear counterstaining. Scale bar = 30 μm. Abbreviations: CXCR4, C-X-C receptor type 4; DAPI, 4′.6-diamidino-2-phenylindole; GSC, glioblastoma stem cell; HSC, hematopoietic stem cell; SDF-1α, stromal-derived factor-1α; SMA, smooth muscle actin.

**Table 1:** Glioblastoma patient demographics.

Patient Characteristics	Thomas et al. [36]	Stupp et al. (Historical Control) [5]
Age (median)	60	57
IDH status:		
Wild-type	90%	95%
Mutant	10%	5%
MGMT status:		
Methylated	45%	45%
Unmethylated	55%	55%
Extent of resection:		
Biopsy	24%	13%
Subtotal resection	28%	33%
Gross total resection	48%	54%
Karnofsky performance score		
≤80	41%	32%
90–100	59%	65%

Molecular scores are calculated among patients with available data.

**Table 2:** Overview of CXCR4 antagonists that have been tested in various types of cancer for their therapeutic power/benefit.

CXCR4 Antagonists	Type of Cancer	References
AMD3100 (plerixafor)	Glioblastoma, breast cancer, cholangiocarcinoma, ovarian cancer, colorectal cancer, melanoma, AML, ALL, CML, MM, non- Hodgkin’s lymphoma, HSC mobilization	[55]
AMD3465	AML, ALL, breast cancer	[55,56,57]
RCP168	AML	[56]
PRX17756	Glioblastoma	[52,53]
BL-8040	Glioblastoma, MM	[54,58]
USL311	Glioblastoma	(ClinicalTrials.gov identifier: NCT02765165), [59]
Balixafortide	Breast cancer	[60]
BKT140	CML, MM	[55,61]
AMD070	Oral cancer	[62]
LY2624587	Non-Hodgkin’s lymphoma, ALL	[63]
T140	CLL, ALL, MM, SCLC	[64,65,66]
TG-0054	CLL, MM	[67]
POL6326	CLL, MM	[67]
MSX-122	CLL, MM, breast cancer	[67,68]
TC14012	CLL	[64]
TN14003	CLL	[64]
CTCE-9908	Ovarian cancer, prostate cancer, esophageal cancer, breast cancer	[69,70,71,72]
CTCE-0021	HSC mobilization	[73,74]
CTCE-0214	HSC mobilization	[55]
ATI-2341	HSC mobilization	[73]

Abbreviations: ALL, acute lymphoblastic leukemia; CLL, chronic lymphocytic leukemia; CML, chronic myelogenous leukemia; HSC, hematopoietic stem cell; MM, multiple myeloma; SCLC, small cell lung cancer.
